# Predicting ROR1/BCL2 combination targeted therapy of small cell carcinoma of the lung

**DOI:** 10.1038/s41419-021-03855-w

**Published:** 2021-06-04

**Authors:** Walter Z. Wang, Konstantin Shilo, Joseph M. Amann, Alyssa Shulman, Mohammad Hojjat-Farsangi, Håkan Mellstedt, Johan Schultz, Carlo M. Croce, David P. Carbone

**Affiliations:** 1 Department of Internal Medicine, Division of Medical Oncology, The Ohio State University, Columbus, OH 43210 USA; 2 The Comprehensive Cancer Center, The Ohio State University, Columbus, OH 43210 USA; 3 Department of Pathology, The Ohio State University, Columbus, OH 43210 USA; 4 Department of Oncology-Pathology, Karolinska Institutet, 17177 Stockholm, Sweden; 5 Kancera AB, Banvaktsvägen 22, 17148 Solna, Sweden; 6 Department of Cancer Biology and Genetics, The Ohio State University, Columbus, OH 43210 USA

**Keywords:** Cancer genetics, Targeted therapies, Small-cell lung cancer

## Abstract

Small cell lung cancer (SCLC) remains a deadly form of cancer, with a 5-year survival rate of less than 10 percent, necessitating novel therapies. Receptor tyrosine kinase-like orphan receptor 1 (ROR1) is an oncofetal protein that is emerging as a therapeutic target and is co-expressed with BCL2 in multiple tumor types due to microRNA coregulation. We hypothesize that ROR1-targeted therapy is effective in small cell lung cancer and synergizes with therapeutic BCL2 inhibition. Tissue microarrays (TMAs) and formalin-fixed paraffin-embedded (FFPE) SCLC patient samples were utilized to determine the prevalence of ROR1 and BCL2 expression in SCLC. Eight SCLC-derived cell lines were used to determine the antitumor activity of a small molecule ROR1 inhibitor (KAN0441571C) alone and in combination with the BCL2 inhibitor venetoclax. The Chou-Talalay method was utilized to determine synergy with the drug combination. ROR1 and BCL2 protein expression was identified in 93% (52/56) and 86% (48/56) of SCLC patient samples, respectively. Similarly, *ROR1* and *BCL2* were shown by qRT-PCR to have elevated expression in 79% (22/28) and 100% (28/28) of SCLC patient samples, respectively. KAN0441571C displayed efficacy in 8 SCLC cell lines, with an IC50 of 500 nM or less. Synergy as defined by a combination index of <1 via the Chou-Talalay method between KAN0441571C and venetoclax was demonstrated in 8 SCLC cell lines. We have shown that ROR1 inhibition is synergistic with BCL2 inhibition in SCLC models and shows promise as a novel therapeutic target in SCLC.

## Introduction

Small cell lung cancer (SCLC) has a 5-year survival rate of only 6 percent, with no approved targeted therapies, underscoring the need for novel therapeutics^1^. While little progress was made over many decades in the treatment of SCLC, in 2019 first-line chemotherapy/immunotherapy for extensive-stage disease was approved^2^. The addition of atezolizumab, a monoclonal antibody targeting programmed death-ligand 1 (PD-L1), to standard chemotherapy increased median overall survival by 2 months to a total of 12.3 months, though objective response rate and median duration of response remained similar between both arms^2^. While this is promising, there is still a great necessity to develop novel and more efficacious treatments for SCLC.

Receptor tyrosine kinase-like orphan receptor 1 (ROR1) is a receptor tyrosine kinase-like protein that has been implicated to play a role in many different types of cancer^3–8^. ROR1 has oncofetal expression, as it is an embryonic protein that is not normally expressed in differentiated cells, but can be re-expressed in cancer cells^9^. Thus, ROR1-targeted therapy could be tumor specific, and a recent study has demonstrated that ROR1 expression is correlated with worsened patient outcomes in lung adenocarcinomas^5^. ROR1 inhibition has been shown to lead to cell death in pancreatic, leukemia, and lung cancer cells, suggesting that it is a viable therapeutic target^4,7,10–13^. Therapies targeting ROR1 have also seen early efficacy for treating hematological malignancies^10,14^.

Interestingly, it has been shown in chronic lymphocytic leukemia (CLL) that the loss of microRNAs (miRNAs) *miR-15a/16-1* is associated with the aberrant expression of ROR1^6,15^. miRNAs are small non-coding RNA molecules that silence the expression of their target mRNAs, acting as negative regulators. Strikingly, the same miRNAs that appear to regulate ROR1 expression in CLL are also responsible for the regulation of the expression of the anti-apoptotic factor BCL2^6^. Recent studies in CLL have shown that an inhibitory antibody targeting ROR1, cirmtuzumab, in combination with the BCL2 inhibitor venetoclax demonstrated synergy, indicating that the combined therapy has a greater clinical impact than either therapy alone^6^. Identification of such mechanism-based drug combinations are commonly used in cancer treatment, with the aims of increasing the therapeutic effect, reducing individual drug dose and subsequent toxicity as well as delaying the onset of drug resistance^16,17^. Drug synergy is critical in the determination of drug combinations, as it is necessary to investigate whether the drugs are working in concert or antagonistically. BCL2 is known to be expressed in SCLC, but targeted monotherapy has not shown significant clinical impact^18^. Examining SCLC samples for co-expression of ROR1 would suggest combination therapies targeting both BCL2 and ROR1 to improve therapeutic effects while reducing treatment-related side effects.

Here, we investigated the prevalence of ROR1 expression in SCLC patient samples and determined the effectiveness of ROR1 inhibition alone and its potential synergy with BCL2 inhibition in SCLC cell lines.

## Methods

### Cell cultures, drugs, and growth assay

SCLC cell lines, H69, H82, H146, H187, H209, H211, H1417, and H1963 were grown in Roswell Park Memorial Institute (RPMI) media with 10% fetal bovine serum (Gibco, Waltham, MA; CN: 11865101). Cell lines were purchased from ATCC (Manassas, VA), authenticated and tested for mycoplasma contamination. Venetoclax was purchased from Selleckchem, Houston, TX (CN: S8048) and KAN0441571C was a gift from Kancera, Solna, Sweden. Cells were plated 12 h prior to start of drug treatment, which lasted for 72 h for all cytotoxicity experiments. Cell viability was estimated by alamarBlue Cell Viability Reagent (ThermoFisher, Waltham, MA; CN: DAL1100) per manufacturer’s instructions.

shRNA knockdown experiments were performed using the pLKO.1-puro vector. To knockdown ROR1 expression, we used ROR1 Mission shRNAs (Sigma, SHCLNG-TRCN0000002024 and SHCLNG-TRCN0000002025) or non-targeting control (Sigma, SHCO16). Lentiviral particles were produced by transfecting 293FT packaging cells with packaging-psPAX2, envelope-pMD2.G and shRNA plasmid as previously described^19^.

### Development of ROR1 tyrosine kinase inhibitor KAN0441571C

The development of the first small molecule inhibitor of the tyrosine kinase ROR1 (KAN0439834) was recently described^10^, and continued development has resulted in a more potent second generation ROR1 inhibitor KAN0441571C^20^.

In brief, a high-throughput screen targeting the tyrosine kinase domain of ROR1 was performed and in the following hit-to-lead and lead optimization stages more than 2000 compounds have been synthesized. The basic scaffold of both KAN0439834 and KAN0441571C was identified in the HTS and was shown to be ATP-competitive, thus the binding site is most probably in the ATP binding pocket of the tyrosine kinase domain. Since the discovery of KAN0439834, approximately 950 additional compounds have been synthesized and tested for cytotoxic effect against primary CLL cells from patients as well as peripheral blood mononuclear cells (PBMC) from healthy donors, leading to the discovery of KAN0441571C. The compound shows a high degree of “druglikeness” as it contains no apparent toxicophores and adheres to criteria typical for an orally active drug (Supplementary Table 1)^20^. The molecular weight is 555 g/mol, the calculated cLogD7.4 is 1.7 and the calculated polar surface area is 73 Å^2^. Compared to the first generation ROR1 inhibitor (KAN0439834), the most important improvements of the second generation compound (KAN0441571C) are a higher cytotoxic potency against various cancer cells in vitro and a substantially longer serum half-life (T1/2) in the mouse (11 h compared to 2.1 h).

### Immunohistochemistry

Tissue microarray (TMA) based samples from surgically resected small cell lung carcinomas (SCLC) were utilized for ROR1 and BCL2 protein expression in human samples. The staining was interpreted as negative (including weak) versus positive (including moderate and strong). IHC staining was performed on paraffin-embedded tissue as previously described^21^. In brief, embedded tissue was cut a 4-μm utilizing positively charged slides, placed in a 60 °C oven for 1 h, cooled, deparaffinized and rehydrated utilizing xylenes and graded ethanol solutions to water. All slides were quenched for 7 min in a 3% hydrogen peroxide aqueous solution to block for endogenous peroxidase. For ROR1 antibody (Rabbit polyclonal, Abcam, ab135669) staining, antigen retrieval was performed by heat-induced epitope retrieval (HIER) in which the slides were placed in a 1× solution of Target Retrieval Solution (Dako, S2367) for 25 min at ≥96 °C using a vegetable steamer (Black & Decker), then cooled for 15 min in solution. Slides were then stained with Dako Link 48 Autostainer Immunostaining System (Agilent Technologies) at room temperature for 60 min, with antibody at a 1:400 dilution. EnVision Flex Mini-Kit HRP (Dako, K8023) was applied for 30 min, then staining was visualized with EnVision Flex DAB-substrate chromagen (Dako, included in EnVision Flex Mini-Kit) applied for 10 min. Slides were counterstained in hematoxylin (Dako/Agilent #SK203), dehydrated through graded ethanol solutions, cleared with xylene, and coverslipped. For BCL2 antibody (Mouse monoclonal, Dako, clone 124), antigen retrieval was performed on-line using Leica’s Bond Epitope Retrieval Solution 2 High pH (ER2, product code AR9640) for 20 min. Primary antibody was incubated for 30 min at room temperature. The detection system used was Leica’s Bond Polymer Refine Detection (product code DS9800). Lastly, sections were incubated with DAB mixed on-line for 10 min. Slides were then counterstained in Leica Bond Hematoxylin, dehydrated through graded ethanol solutions, cleared with xylene and coverslipped.

### Quantitative RT-PCR

Total RNA was isolated using the miRNeasy Kit (Qiagen, Hilden, Germany; CN: 217084) or miRNeasy FFPE Kit (Qiagen; CN: 217504) according to the manufacturer’s instructions. Synthesis of cDNA from total RNA (100 ng) was performed using a High-Capacity cDNA Reverse Transcription Kit following manufacturer’s instructions (Applied Biosystems, Foster City, CA; CN: 4368814). Reverse transcription thermo cycling parameters were 25 °C for 10 min, 37 °C for 120 min and 85 °C for 5 min for mRNA, or 16 °C for 30 min, 42 °C for 30 min and 85 °C for 5 min for miRNA. Reactions were performed on a MyCycler (Bio-Rad, CA, USA). Quantitative real-time PCR was performed in triplicate using the Viia7 system in 10 μl reaction volumes containing 5 μl of PCR master mix (Taqman 2× Universal PCR master mix, Applied Biosystems, CN: 4324018) each primer-probe 0.5 μl, 3.5 μl of nuclease-free water, and 1 μl of cDNA (total 10 μl) in optical 384-well plates (Applied Biosystems). qPCR cycling conditions were: 95 °C for 10 min, followed by 40 cycles of 95 °C for 15 s and 60 °C for 60 s. Triplicate qPCR reactions were performed for each cDNA sample for all experiments. The threshold fluorescence level was set automatically for each plate using QuantStudio Real-Time PCR Software (Applied Biosystems). All probes (ROR1: Hs00938677_m1, CN: 4331182; BCL2: Hs00608023_m1, CN: 4331182; GAPDH: Hs02786624_g1, CN: 4331182) utilized were TaqMan Gene Expression assays purchased from ThermoFisher Scientific. Human Lung Total RNA used as a control was purchased from ThermoFisher Scientific. Results were normalized using GAPDH expression levels. Correlation analyses were conducted using Spearman’s rank correlation coefficient in the R programming language.

### Statistical analysis

Statistical analyses were performed using Graphpad Prism version 8. Differences were considered significant when the *p* values were less than 0.05.

### Synergy analysis

We obtained dose curves for venetoclax and KAN0441571C as single agents and in constant ratios of their IC50 values to determine to what degree these agents showed synergy. Combination index (CI) scores were calculated as previously described utilizing CompuSyn software (ComboSyn, Inc), which uses the Chou-Talalay combination index method based on the principles of the median-effect equation^16^. Synergy of two agents was defined as CI < 1, additivity as 1<CI < 1.5, and antagonism as CI > 1.5^22^. Isobolograms were also drawn utilizing CompuSyn to visualize synergy. In brief, the amount of each individual drug needed to achieve an effect of 50, 75, and 90 percent is calculated and used as intercepts to generate an isobole connecting those points. Dose pairs for the combination therapy are then plotted, with points below the isobole are considered synergistic, on the isobole additive, and above the isobole antagonistic^17^.

### Western blotting

Western blot analysis was performed following standard procedures. Whole-cell protein lysates were prepared using RIPA buffer with cOmplete^TM^ Mini protease inhibitor (Sigma, CN: 11836170001) and PhosSTOP (Sigma, CN: 4906845001). Protein concentrations were assayed using the Pierce BCA assay (ThermoFisher, CN: 23225), then equal amounts of protein were suspended in SDS loading buffer with DTT, separated on Criterion TGX gels 4–15% SDS-PAGE gels (BioRad, CN: 5671084), and transferred onto PVDF membrane using the Transblot Turbo (BioRad) system. The membranes were then treated with appropriate antibodies according to the methods recommended by their manufacturer and LiCor, then imaged on a LiCor CLx imager. The following antibodies were used in this study: ROR1 (1:1000 dilution, CN: D6T8C), BCL2 (1:1000 dilution, CN: 15071) and β-actin (1:1000 dilution, CN: 3700) from Cell Signaling Technologies. Phopho-ROR1 antibody (1:250 dilution) was a gift from Kancera, and was produced as previously described^10^.

## Results

### ROR1 and BCL2 are co-expressed in patient samples of SCLC

**Fig. 1:**
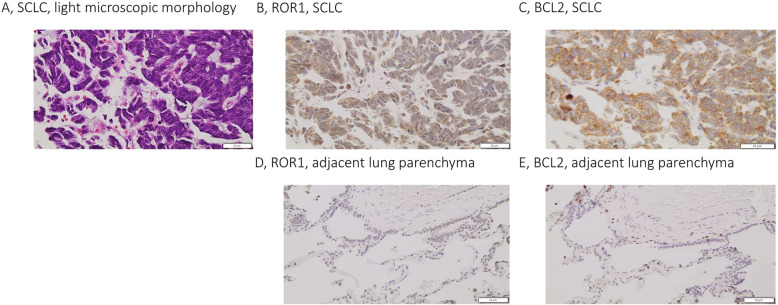
ROR1 and BCL2 expression in small cell lung carcinoma (SCLC). Light microscopic morphology of a representative case of SCLC (**A**) showing co-expression of ROR1 (**B**) and BCL2 (**C**) in the same tissue core of a tissue microarray; original magnification x400. No expression of ROR1 and BCL2 is observed in non-neoplastic lung parenchyma stained for ROR1 (**D**) and BCL2 (**E**); the same tissue core, original magnification x400.

To investigate SCLC for ROR1 and BCL2 co-expression, we evaluated tissue microarray-based samples from SCLC patients by immunohistochemistry. SCLC showed frequent diffuse cytoplasmic expression of ROR1 and BCL2 (Fig. 1). Positive expression was defined by scoring IHC staining on a scale of 0–3+, with representative images shown (Supplementary Fig. 1). ROR1 expression was identified in 93% (52/56) of SCLC tumors, while BCL2 expression was identified in 86% (48/56) of SCLC tumors, and 83% (45/56) of SCLC tumors showed expression of both ROR1 and BCL2 in the same tumor cells. It was observed that in positive samples all tumor cells stained for ROR1 and BCL2 expression. This confirms that a majority of SCLC express ROR1 and BCL2, suggesting that a ROR1-targeted therapy could be effective in SCLC patients alone or in combination with BCL2 inhibition (Table 1).

**Table 1:** BCL2 and ROR1 expression in SCLC IHC samples.

BCL2 Status	ROR1 Status		Total
	Positive	Negative	
Positive	45 (83.36%)	3 (5.36%)	48 (85.71%)
Negative	7 (12.50%)	1 (1.79%)	8 (14.29%)
Total	52 (92.86%)	4 (7.14%)	

**Fig. 2:**
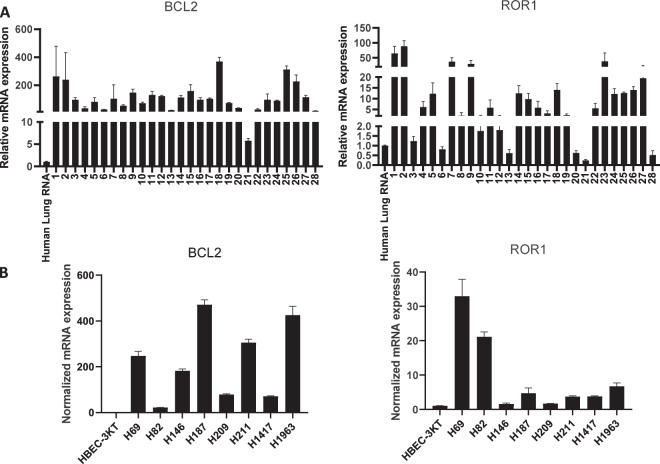
RT-qPCR analysis of *BCL2* and *ROR1* expression in SCLC. **A** Analysis of 28 SCLC FFPE samples with Human Lung RNA used as normal control. **B** Analysis of 8 SCLC cell lines with HBEC-3KT used as normal control. All gene expression levels were normalized to GAPDH.

We then extracted RNA from 28 formalin-fixed paraffin-embedded (FFPE) SCLC patient biopsy samples to analyze for *ROR1* and *BCL2* expression via RT-qPCR to corroborate the results seen via IHC. Of these samples, 82 percent (23/28) were stage 3–4 and 11 percent (3/28) were treated prior to biopsy (Supplementary Table 1). Results were compared to Human Lung Total RNA as a normal control, and elevated expression of *ROR1* and *BCL2* was defined as the relative mRNA expression of >2 compared to control, with results being normalized to GAPDH expression levels. We found that 79 percent (22/28) of the samples had elevated *ROR1* expression compared to normal, and 100 percent (28/28) of samples had elevated *BCL2* expression (Fig. 2A). Of those, 79 percent (22/28) had both elevated *ROR1* and *BCL2* expression, which correlates well with what was observed in the TMA analysis. To further validate these results, we utilized published SCLC sequencing data from 81 patient samples to determine that 92 percent (78/81) and 100 percent (81/81) of samples expressed ROR1 and BCL2, respectively^23^. Active gene expression was defined as having a zFPKM score of greater than −3 as defined in the previous studies^24^. These data corroborate our observations of ROR1 and BCL2 expression via IHC and RT-qPCR. To investigate the role of miRNA-15/16 in the regulation of *ROR1* and *BCL2* expression in SCLC, we performed RT-qPCR to determine the expression of *miR-15a*, *miR-15b*, and *miR-16* in these SCLC FFPE samples. Their expression levels are plotted against each other, as well as *BCL2* and *ROR1* relative expression (Supplementary Fig. 2). We found that there was a positive correlation between the expression of the miRNAs with each other, as well as between *BCL2* and *ROR1* expression. Additionally, we determined that there is a negative correlation between the expression of the miRNAs and the expression level of *BCL2* and *ROR1*, suggesting that the expression of these miRNAs may act as a negative regulator of *ROR1* and *BCL2* expression in SCLC as previously described in CLL^6,25^.

### Single agent ROR1 inhibition, but not BCL2 inhibition alone, is effective in in vitro models of SCLC

**Fig. 3:**
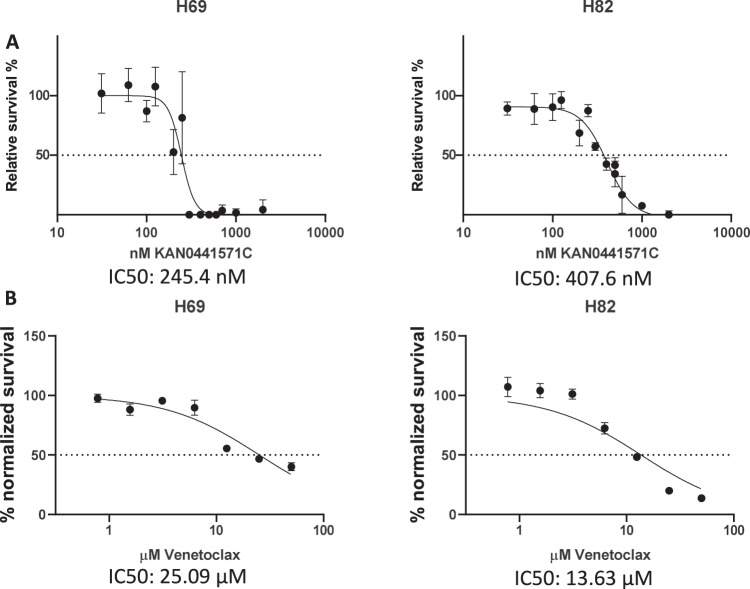
Dose–response curves of the ROR1 inhibitor KAN0441571C and BCL2 inhibitor venetoclax in representative cell lines H69 and H82 via alamarBlue Cell Viability Reagent. **A** KAN0441571C dose–response curves and relative IC50 are aggregated results from 2 experiments. **B** Venetoclax dose–response curves and IC50.

In order to investigate the efficacy of ROR1 inhibition in vitro, we characterized 8 SCLC cell lines (H69, H82, H146, H187, H209, H211, H1417, and H1963) for *ROR1* and *BCL2* expression (Fig. 2B), normalizing gene expression to GAPDH. Of these, all 8 had elevated *ROR1* and *BCL2* expression compared to HBEC-3KT cells, which are immortalized human bronchial epithelial cells^26^. H146 cells had the lowest expression level of *ROR1*, with a relative mRNA expression of a factor of approximately 2 compared to HBEC-3KT control. As these results are consistent with the data shown in human samples, we used these SCLC cell lines to test the efficacy of ROR1 and BCL2 inhibition in vitro. For each cell line we generated dose–response curves of a novel small molecule ROR1 inhibitor, KAN0441571C (Fig. 3A and Supplementary Fig. 3), as well as a BCL2 inhibitor, venetoclax (ABT-199) (Fig. 3B and Supplementary Fig. 4), to determine the relative cell viability and the IC_50_ of each drug. BCL2 inhibition via venetoclax as a single agent showed limited efficacy in the SCLC cell lines, with an IC_50_ of 16.7 μM on average. In contrast, KAN0441571C as a single agent showed efficacy at lower levels, with an IC_50_ of 299 nM on average (Supplementary Table 2). These results suggest that ROR1 may be a viable target for SCLC therapy, even as a monotherapy.

### KAN0441571C and venetoclax synergize as a combination therapy against SCLC cell lines

**Fig. 4:**
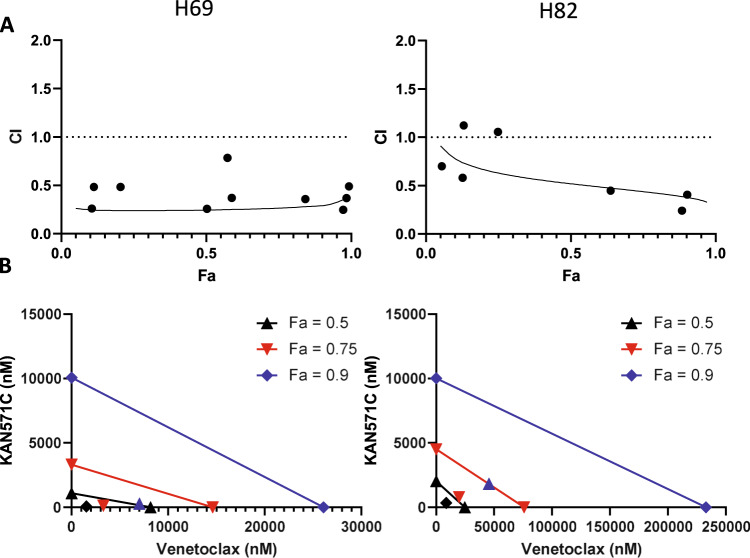
KAN0441571C and venetoclax display synergy in causing SCLC cell line death. **A** Combinatorial index (CI) values across F_a_ in H69 and H82 cells, where Fa is percentage cell death. Synergy is defined by CI < 1, additivity 1 <CI < 1.5, antagonism CI > 1.5. Combo2 points in H69 graph are from an additional experiment combined with the initial experiment due to initial points being clustered at high F_a_ values. **B** Isoboles at 3 Fa values in H69 _a_nd H82 cells, where Dose A is defined as KAN0441571C and Dose B is venetoclax. Synergy is defined by a combination point below the isobole, additivity a point on the isobole, and antagonism a point above the isobole.

We next wanted to determine whether ROR1-targeted therapy and BCL2 inhibition would demonstrate synergy. Here, we determined synergism utilizing calculated combination index (CI) values via the Chou-Talalay method derived from the mass-action law, as well as isoboles to quantitatively determine synergy between KAN0441571C and venetoclax. A constant ratio of 1:50 KAN0441571C to venetoclax was chosen for these experiments from the average IC_50_ value of each individual drug. F_a_ was defined as percent cell death with 1 indicating complete cell death, Dose A as KAN0441571C, Dose B as venetoclax, and Combo as the combined treatment of KAN0441571C and venetoclax. We determined synergy in all eight tested SCLC cell lines, though some had additivity at lower F_a_ value (Fig. 4A and Supplementary Fig. 5). For H69 cells, due to the initial experimental points being clustered in high F_a_ values, an additional experiment was performed utilizing a 1:1 ratio of KAN0441571C to venetoclax, which resulted in additional points that fit well along the initially calculated CI values. In isobole analysis, nearly all cell lines demonstrated synergy at F_a_ values of 0.5, 0.75, and 0.9 (Fig. 4B and Supplementary Fig. 6). These data indicate that ROR1-targeted therapy synergizes with BCL2 inhibition across a range of SCLC cell lines and would demonstrate synergy and suggest potential efficacy in treating SCLC patients.

### Synergy between venetoclax and KAN0441571C is not due to off-target effects of KAN0441571C

Since KAN0441571C is a novel candidate drug, we wanted to be certain that the drug was inhibiting the intended target, ROR1. First, we demonstrated that treating H69 cells with KAN0441571C decreased phospho-ROR1 levels in a dose-dependent manner upon Western blot analysis (Supplementary Fig. 7). Furthermore, we show that levels of phospho-ERK, a downstream effector of ROR1 signaling, also decreased in a dose-dependent manner 30 min after KAN0441571C application in both H69 and H82 cell lines, and that levels of both total and active (non-phosphorylated) β-catenin levels decrease over a time course of 3 days after 1 μM KAN0441571C treatment (Supplementary Fig. 8). These findings reflect previously published data that demonstrated ROR1 inhibition decreased canonical Wnt-signaling through inactivation of β-catenin, dephosphorylated Src, and inhibited the PI3K/AKT/mTOR pathway^10,12,20^. We then performed shRNA-mediated knockdown of ROR1 in H69 and H82 cell lines and noted a significant decrease in relative cell survival after 72 h of growth compared to untreated parental controls and non-targeting control (NTC). With the addition of 25 μM venetoclax, the calculated cell viability IC_50_ of venetoclax for H69 cells, we noted a significant decrease in relative survival compared to ROR1 shRNA alone (Supplementary Fig. 9). These data suggest that the effects we are observing with KAN0441571C are on-target.

As an additional test for specificity, we performed KINOMEscan™ profiling (DiscoverX, San Diego, CA, USA) to identify off-target effector kinases of KAN0441571C, followed by determination of potencies in a kinase activity assay (Flashplate-based radiometric ^33^P assay; ProQinase, Freiburg, Germany). According to these assays, the kinases that have the greatest potential to be off-targets for KAN0441571C are CDK4, CDK6, and CDK9, which could be responsible for the effects and synergy demonstrated by KAN0441571C and venetoclax (Supplementary Table 3). To investigate whether the inhibition of these potential off-target kinases CDK4 and CDK9 is responsible for the synergy between KAN0441571C and venetoclax, we utilized the CDK4/6 inhibitor abemaciclib and the CDK9 inhibitor SNS-032 in combination with venetoclax in H69 and H82 cells (Supplementary Fig. 10). Cells were treated with a ratio of 1:5 of abemaciclib or SNS-032 to venetoclax based on preliminary IC_50_ values. What we observed in these two cell lines was mostly additive. With SNS-032, slight synergy with venetoclax was observed at high F_a_ in H69 cells, but not at the levels seen with KAN0441571C. In H82 cells, SNS-032 demonstrated only additivity with venetoclax. Abemaciclib showed no synergy with venetoclax in either cell line, as we obtained CIs between additivity and antagonism in both. These results indicate that the synergy found between KAN0441571C and venetoclax is a result of on-target ROR1 specific inhibition, rather than modulation or inhibition of another kinase.

## Discussion

Our study shows that ROR1 is commonly expressed in SCLC, and that it is a potential therapeutic target that would have broad therapeutic value to SCLC patients in a targeted, tumor-specific manner. We demonstrate the efficacy of a novel, small molecule ROR1 inhibitor, KAN0441571C, in SCLC in vitro. Novel small molecule targeted therapies are urgently needed in SCLC, as none are currently available to supplement the current standard-of-care of chemotherapy with or without monoclonal antibodies against programmed death-ligand 1 (PD-L1). This standard therapy has significant systemic toxicities, and almost all patients relapse eventually, needing other therapy options. ROR1-targeted therapy is expected to be more cancer-specific than chemotherapy, with fewer side effects due to its oncofetal expression. Cirmtuzumab, an inhibitory monoclonal antibody targeting ROR1, has already undergone a Phase I clinical trial in CLL patients with the conclusion that it is safe and well tolerated^14^. ROR1 chimeric antigen receptor-specific autologous T-lymphocytes (CAR-T) therapy is undergoing clinical trials in triple-negative breast cancers (TNBC) and non-small cell lung cancers (NSCLC)^14,27^. With ROR1-targeted therapy already showing promising signs of activity in various cancer types, KAN0441571C brings the added benefits of being a small molecule inhibitor that directly inhibits the tyrosine kinase domain of a selectively overexpressed kinase, ROR1. This makes it more likely that KAN0441571C will have increased bioavailability and better penetration into solid tumors, as well as more easily cross the blood-brain barrier compared to larger therapeutic molecules^28^.

Additionally, our study demonstrated that most tumors co-express higher than normal amounts of BCL2 and ROR1 compared to normal controls, which may be at least in part due to lower levels of *miR-15/16*. The fact that these two proteins are both overexpressed in SCLC led us to examine the combination of a targeted ROR1 inhibitor and a BCL2 inhibitor. We noted a marked synergy between ROR1-targeted therapy and BCL2 inhibition utilizing the FDA-approved inhibitor venetoclax, a drug that has demonstrated efficacy in other malignancies such as CLL and acute myeloid leukemia (AML) as well as preliminary efficacy in SCLC models with high levels of BCL2 expression^29–32^. While there is some concern regarding dose-limiting toxicities such as tumor lysis syndrome in hematologic malignancies such as CLL with venetoclax therapy, no such adverse events have been observed in ongoing solid tumor safety and efficacy trials and there were no discontinuations of therapy due to adverse events^33^. Furthermore, initial reports suggest that venetoclax in conjunction with other therapies, such as tamoxifen, is well-tolerated by patients and demonstrates promising results in tumor treatment^33^. As BCL2 is an actionable target with an FDA-approved targeted therapy, we believe that the synergy we’ve observed with KAN0441571C and BCL2 inhibitors in SCLC would be of potential clinical benefit. The combination of KAN0441571C and venetoclax is also supported by a recent study showing that there is increased killing efficacy of ROR1-expressing diffuse large B-cell lymphoma (DLBCL) cell lines with the combination therapy compared to either drug alone^20^. Compared to current standard-of-care immunotherapy plus cisplatin/etoposide chemotherapy, two targeted cancer-specific therapies could have reduced toxicity and provide options for patients relapsing on standard of care therapy. We feel that this combination warrants further investigation to determine its efficacy in SCLC patients.

ROR1 expression has also been associated with cancer stem-like cells (CSCs) as well as those with metastatic potential, and its inhibition has been shown to decrease stem cell markers in CLL^7,14,34^. It is possible that KAN0441571C and venetoclax demonstrate synergy by targeting separate cell populations in SCLC, with KAN0441571C killing CSCs and venetoclax eliminating the bulk tumor. SCLC has been shown to have significant intratumoral heterogeneity, with a stem-like drug-resistant population supporting a faster proliferating bulk tumor population^35^. These stem-like cells lack the expression of achaete-scute family BHLH transcription factor 1 (ASCL1), a critical transcription factor in SCLC that is known to drive the expression of BCL2. However, these cells could be ROR1 positive, especially as ROR1 is normally an embryonically expressed protein^36,37^. Resistance and subsequent repopulation by these CSCs could be the root cause of therapy failure, making the elimination of CSCs a necessity for durable and effective treatment. The combination of ROR1 and BCL2 inhibition could target both of these populations and may represent a promising advance in SCLC therapy options.

## Supplementary information

Supplementary Material
