# Supplementary material for: Predicting ROR1/BCL2 combination targeted therapy of small cell carcinoma of the lung
Source: Cell Death Dis. 2021 Jun 4;12(6):577. doi: 10.1038/s41419-021-03855-w (PMC8178315; doi:10.1038/s41419-021-03855-w)
Supplement: Supplementary file 1 — Supplementary Material [file 41419_2021_3855_MOESM1_ESM.pptx]

## Slide 1
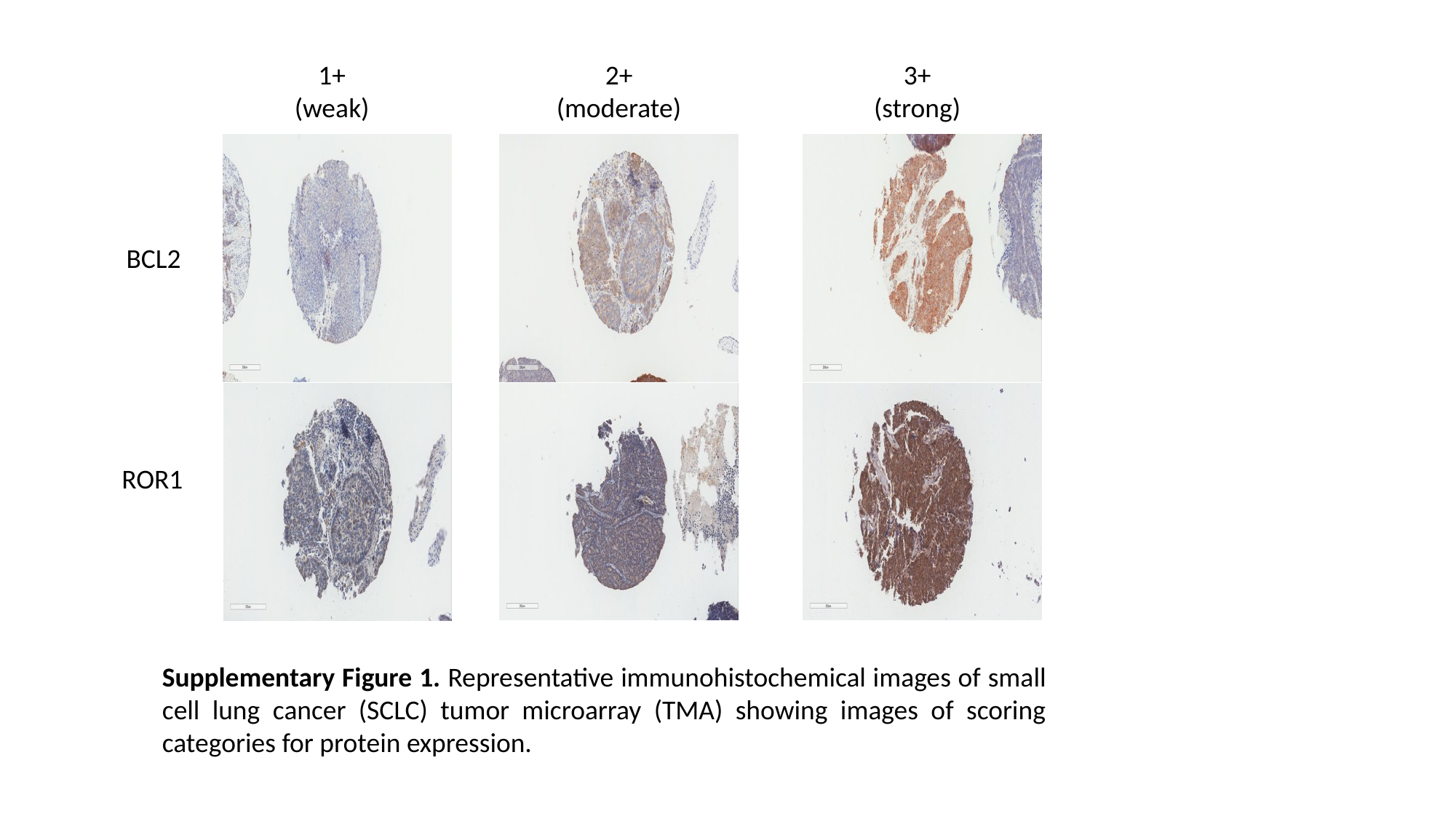

1+
(weak)
2+
(moderate)
3+
(strong)
BCL2
ROR1
Supplementary Figure 1. Representative immunohistochemical images of small cell lung cancer (SCLC) tumor microarray (TMA) showing images of scoring categories for protein expression.

## Slide 2
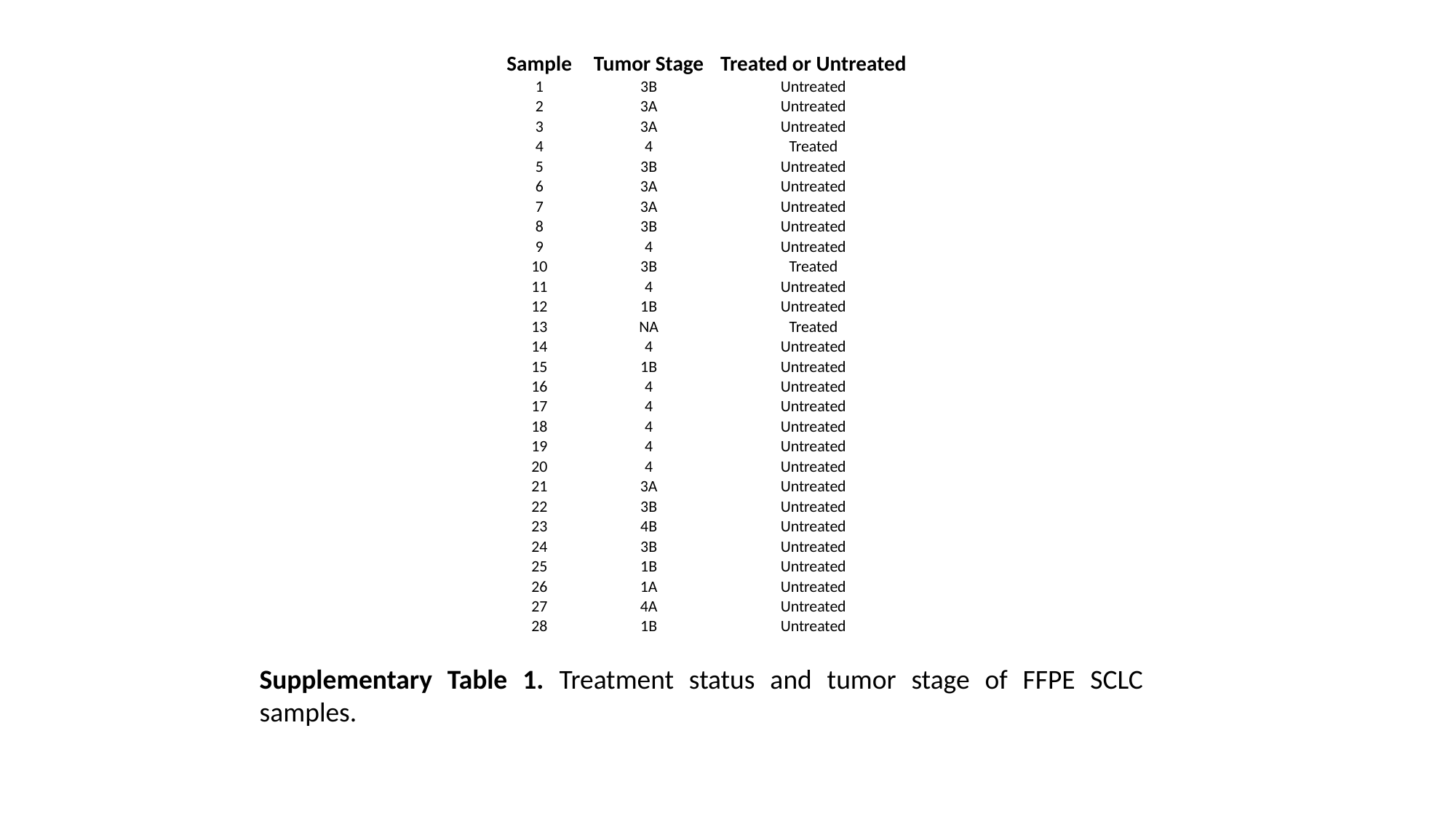

| Sample | Tumor Stage | Treated or Untreated |
| --- | --- | --- |
| 1 | 3B | Untreated |
| 2 | 3A | Untreated |
| 3 | 3A | Untreated |
| 4 | 4 | Treated |
| 5 | 3B | Untreated |
| 6 | 3A | Untreated |
| 7 | 3A | Untreated |
| 8 | 3B | Untreated |
| 9 | 4 | Untreated |
| 10 | 3B | Treated |
| 11 | 4 | Untreated |
| 12 | 1B | Untreated |
| 13 | NA | Treated |
| 14 | 4 | Untreated |
| 15 | 1B | Untreated |
| 16 | 4 | Untreated |
| 17 | 4 | Untreated |
| 18 | 4 | Untreated |
| 19 | 4 | Untreated |
| 20 | 4 | Untreated |
| 21 | 3A | Untreated |
| 22 | 3B | Untreated |
| 23 | 4B | Untreated |
| 24 | 3B | Untreated |
| 25 | 1B | Untreated |
| 26 | 1A | Untreated |
| 27 | 4A | Untreated |
| 28 | 1B | Untreated |
Supplementary Table 1. Treatment status and tumor stage of FFPE SCLC samples.

## Slide 3
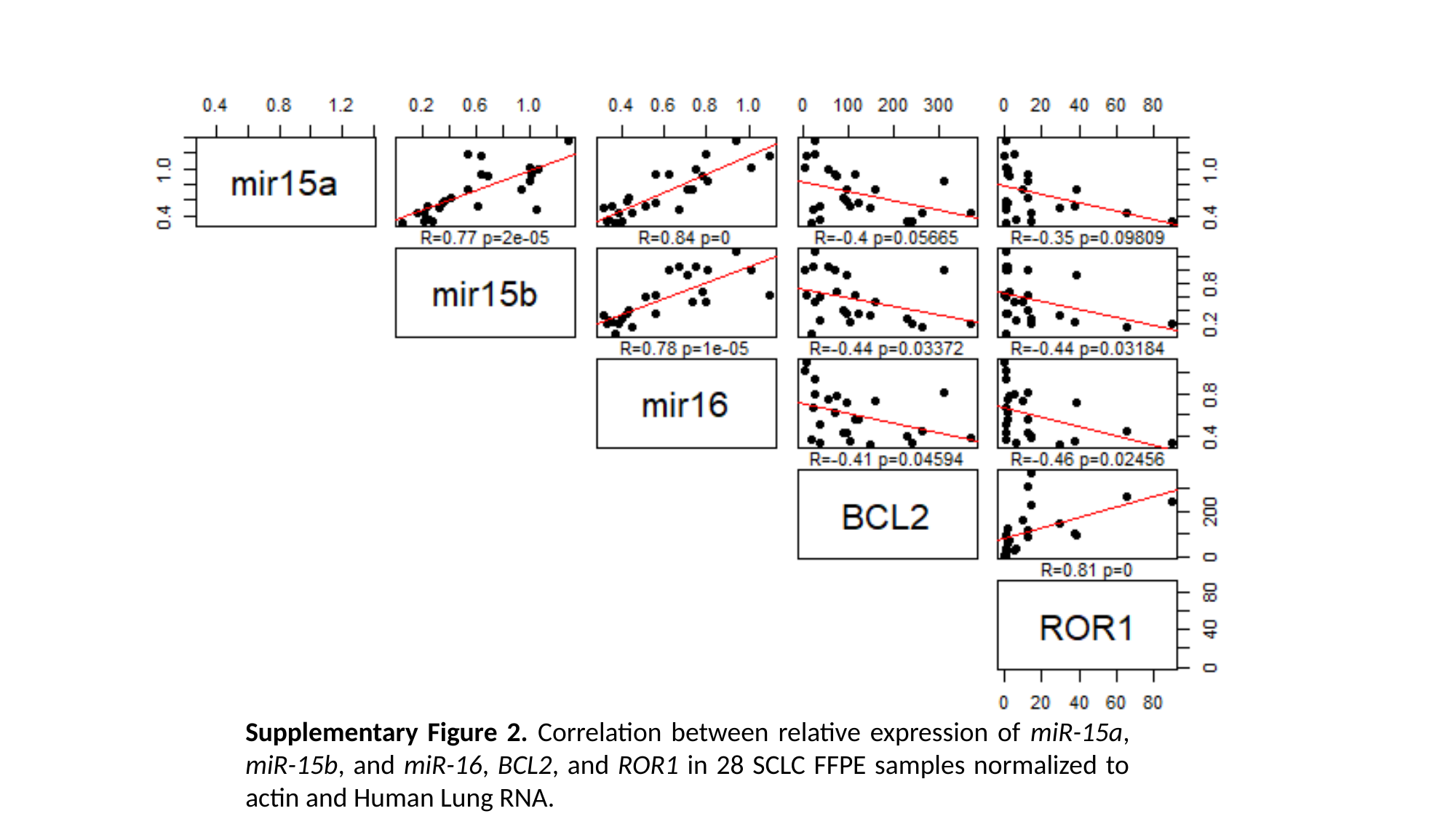

Supplementary Figure 2. Correlation between relative expression of miR-15a, miR-15b, and miR-16, BCL2, and ROR1 in 28 SCLC FFPE samples normalized to actin and Human Lung RNA.

## Slide 4
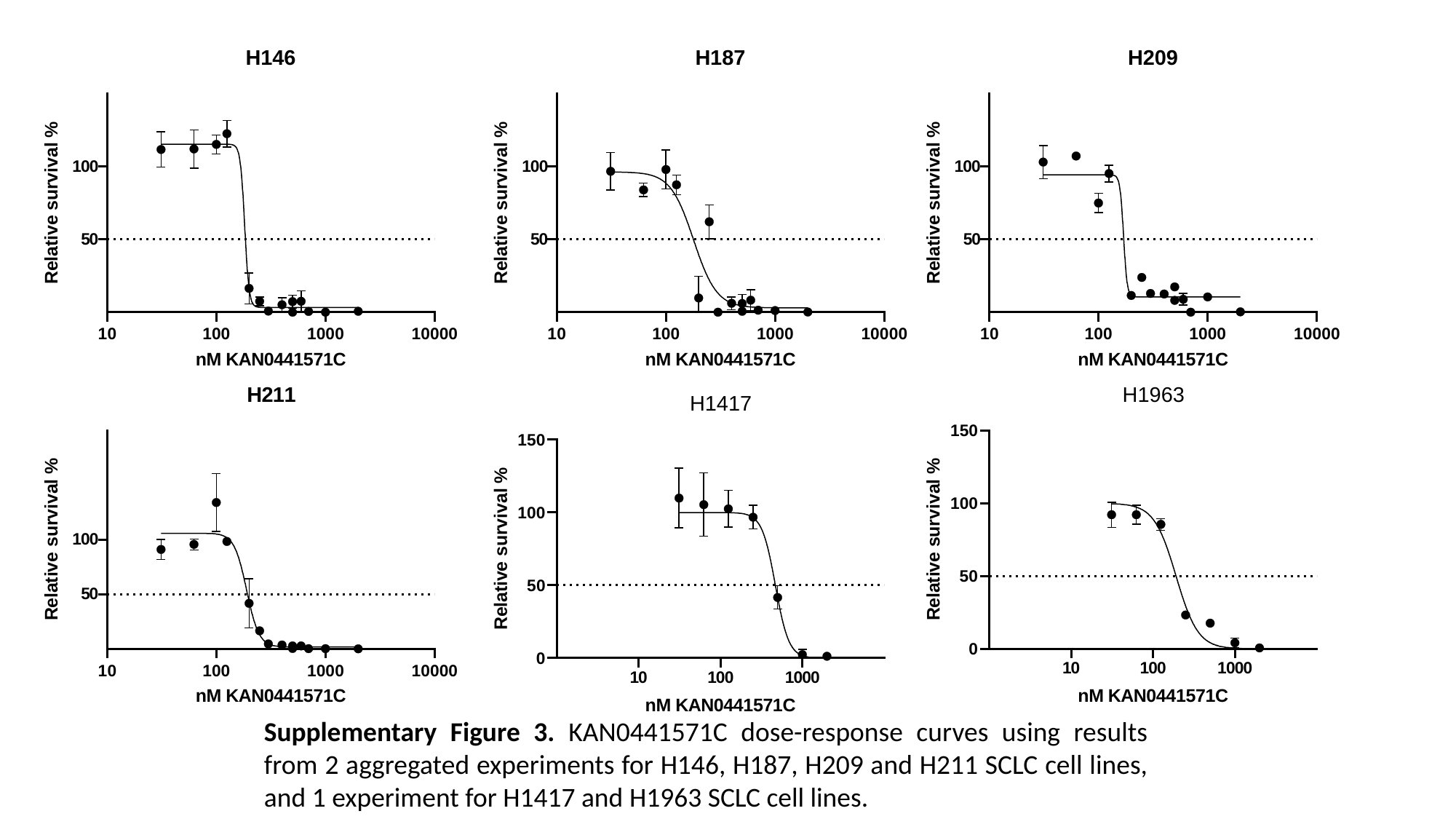

Supplementary Figure 3. KAN0441571C dose-response curves using results from 2 aggregated experiments for H146, H187, H209 and H211 SCLC cell lines, and 1 experiment for H1417 and H1963 SCLC cell lines.

## Slide 5
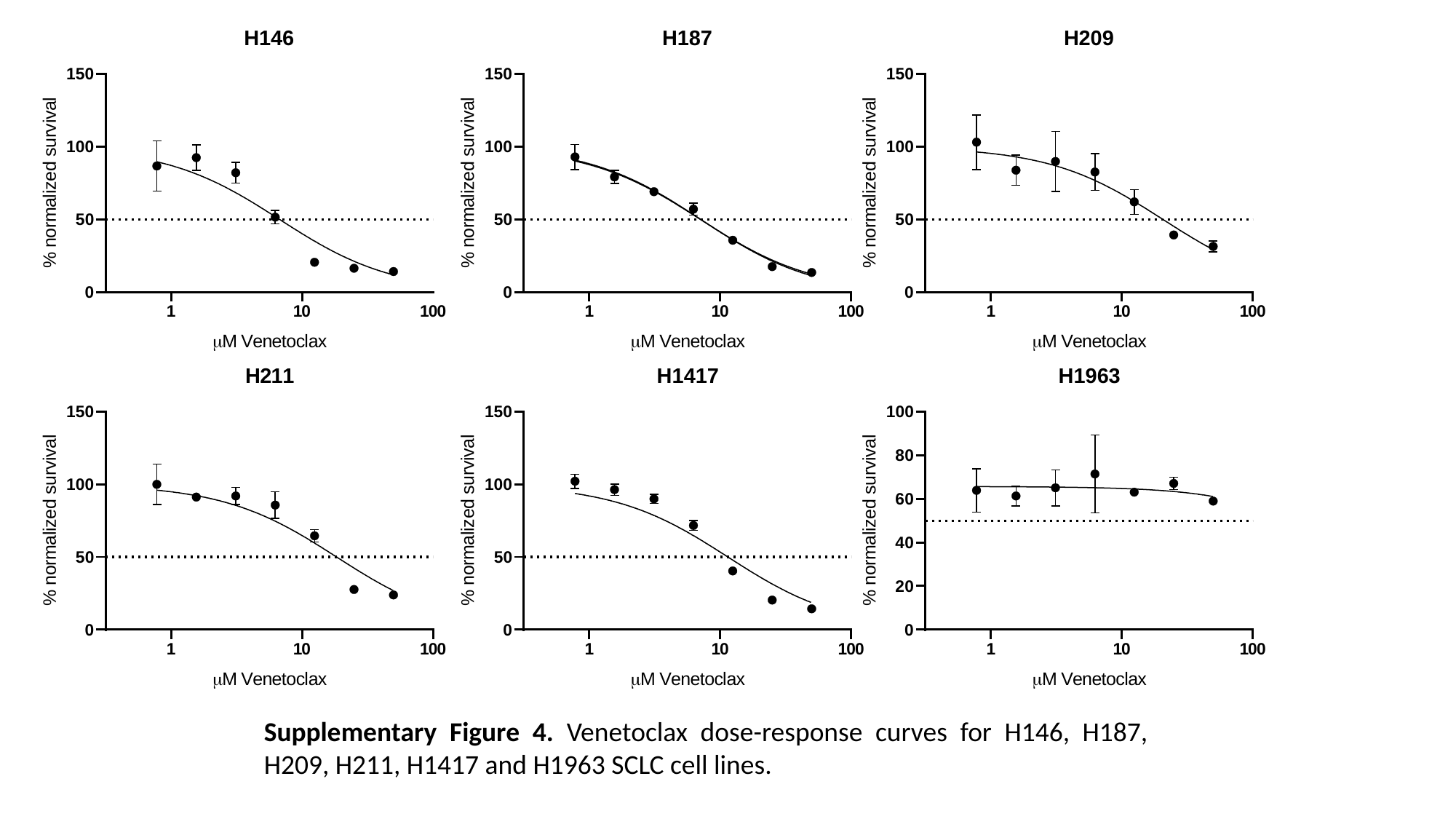

Supplementary Figure 4. Venetoclax dose-response curves for H146, H187, H209, H211, H1417 and H1963 SCLC cell lines.

## Slide 6
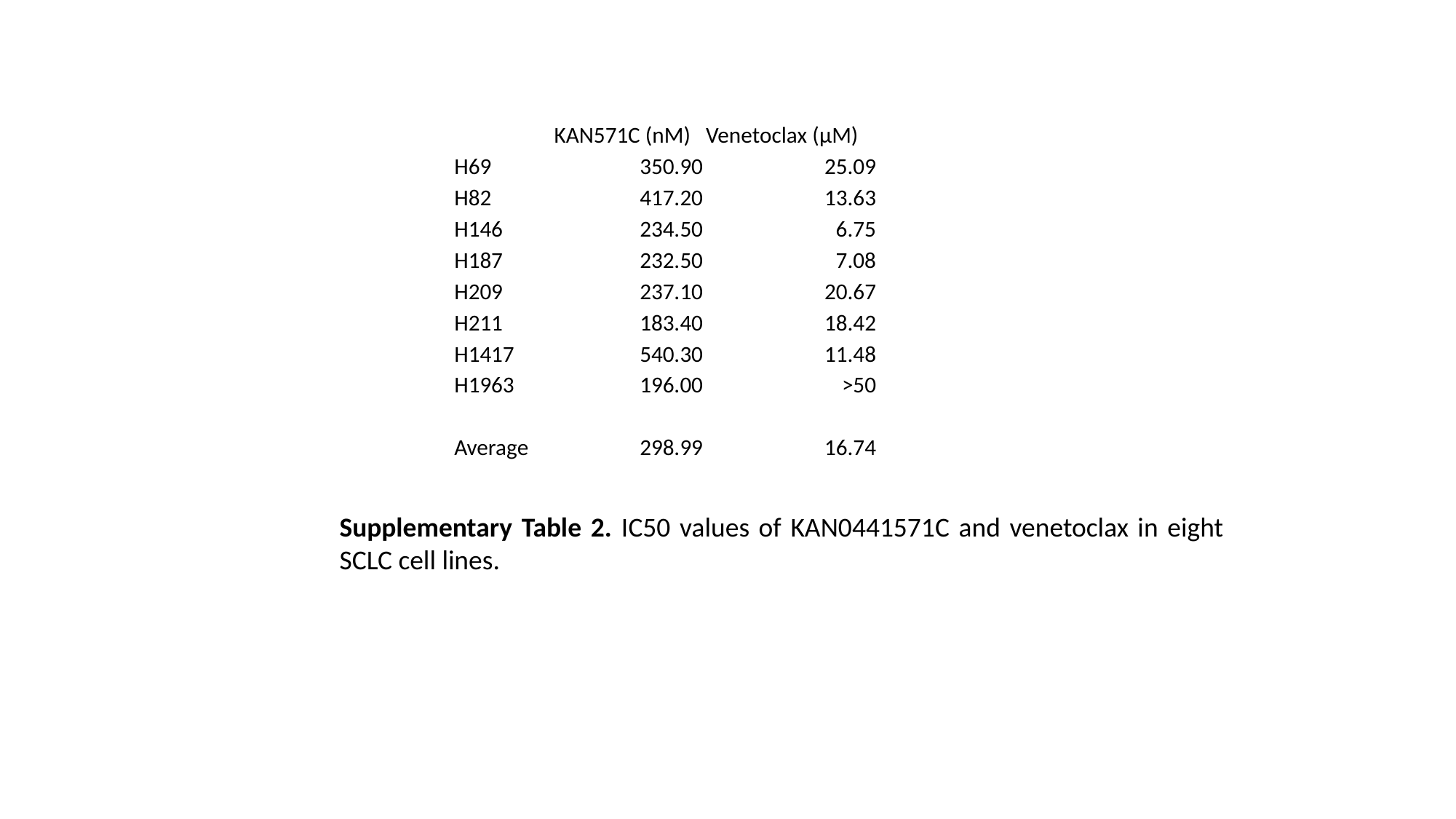

| | KAN571C (nM) | Venetoclax (μM) |
| --- | --- | --- |
| H69 | 350.90 | 25.09 |
| H82 | 417.20 | 13.63 |
| H146 | 234.50 | 6.75 |
| H187 | 232.50 | 7.08 |
| H209 | 237.10 | 20.67 |
| H211 | 183.40 | 18.42 |
| H1417 | 540.30 | 11.48 |
| H1963 | 196.00 | >50 |
| | | |
| Average | 298.99 | 16.74 |
Supplementary Table 2. IC50 values of KAN0441571C and venetoclax in eight SCLC cell lines.

## Slide 7
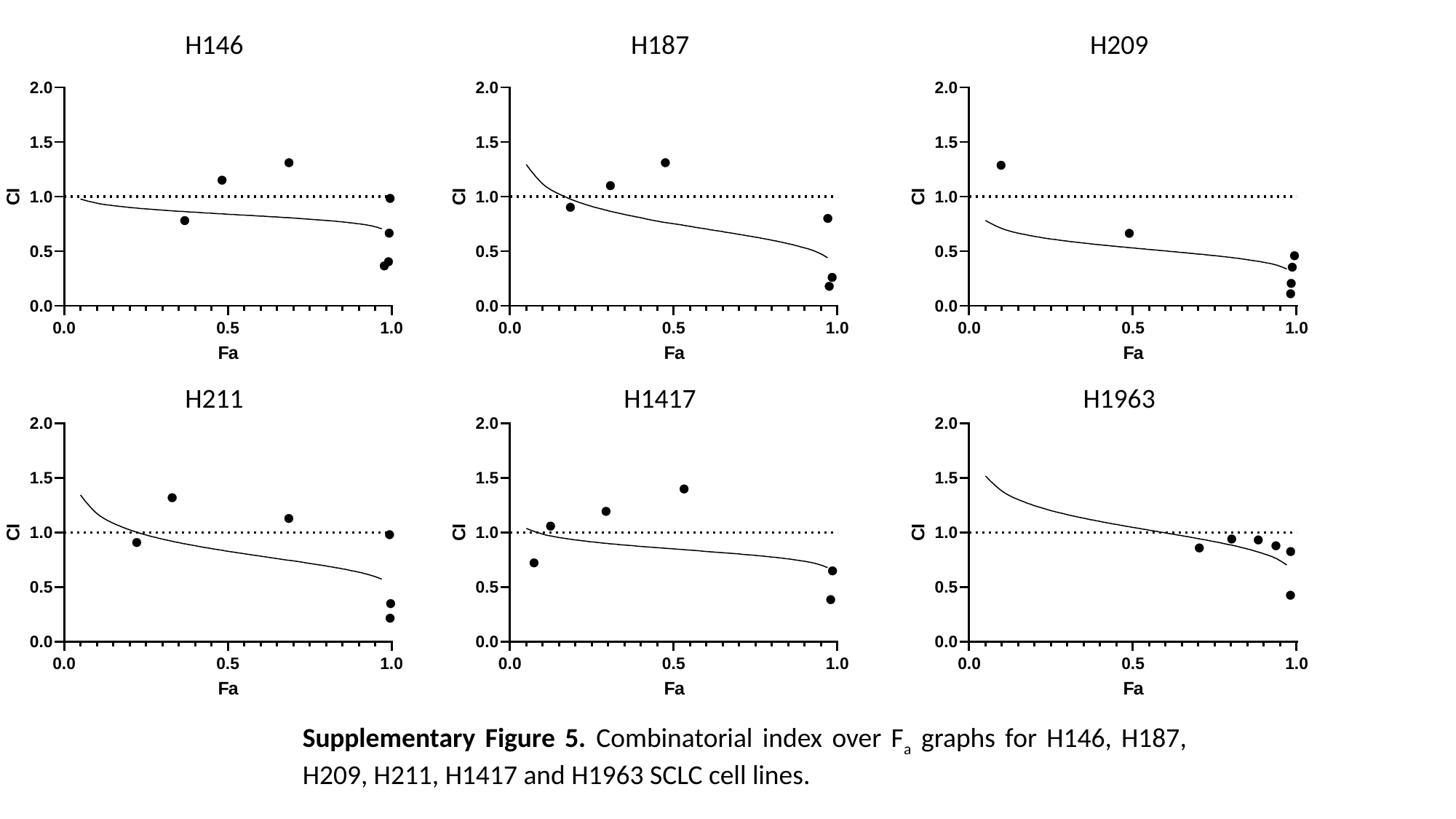

H146
H187
H209
H211
H1417
H1963
Supplementary Figure 5. Combinatorial index over Fa graphs for H146, H187, H209, H211, H1417 and H1963 SCLC cell lines.

## Slide 8
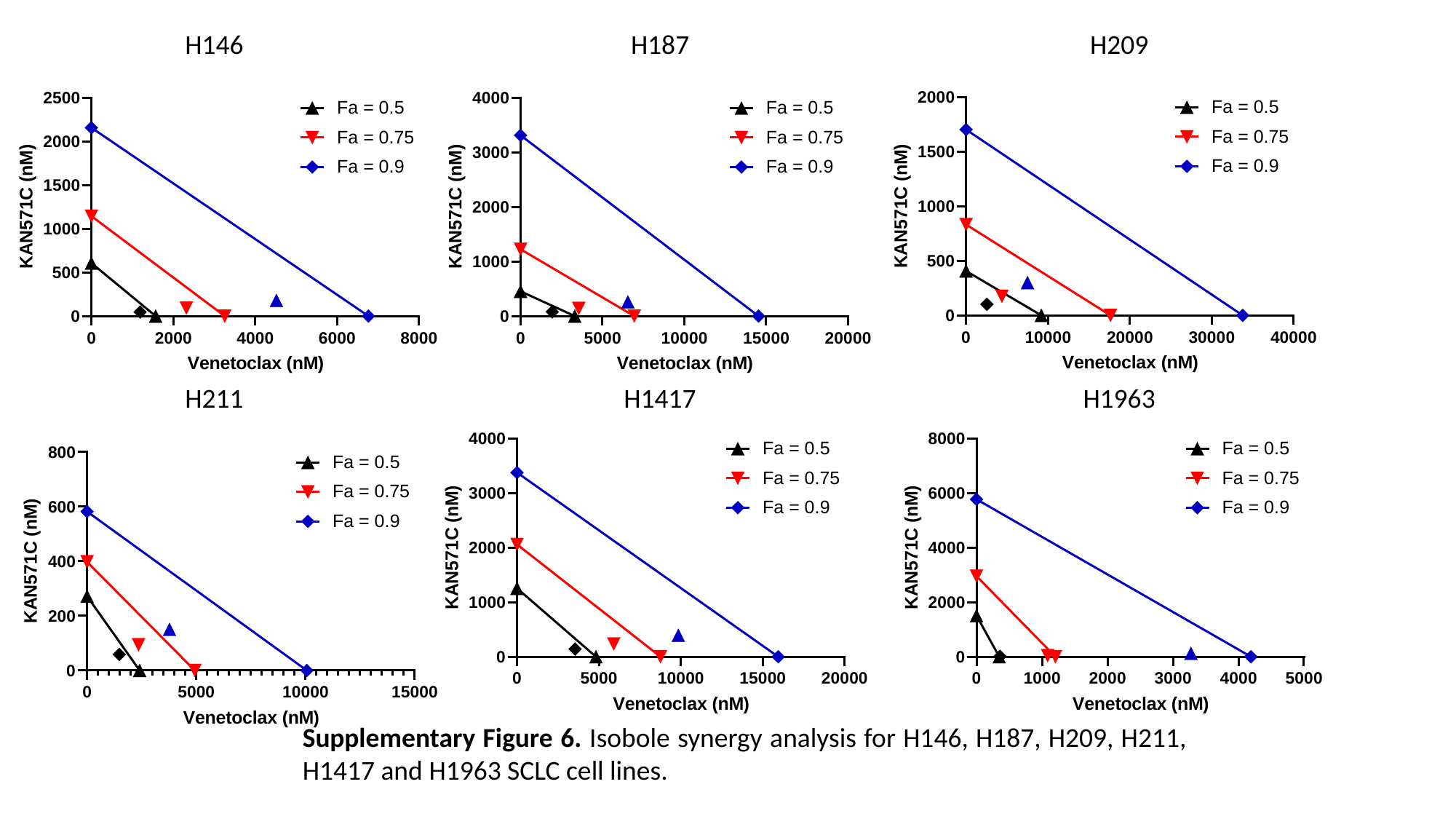

H146
H187
H209
H211
H1417
H1963
Supplementary Figure 6. Isobole synergy analysis for H146, H187, H209, H211, H1417 and H1963 SCLC cell lines.

## Slide 9
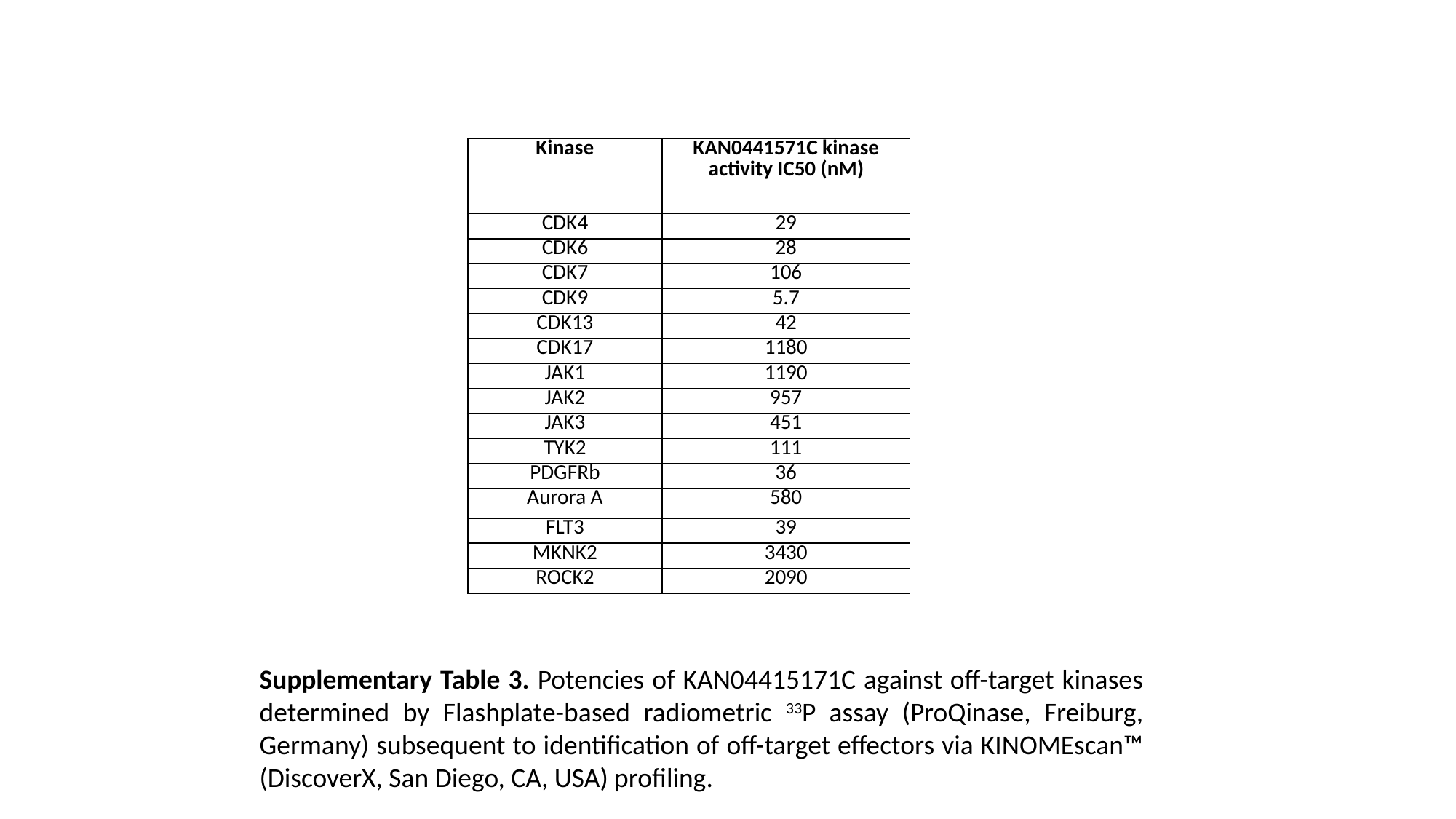

| Kinase | KAN0441571C kinase activity IC50 (nM) |
| --- | --- |
| CDK4 | 29 |
| CDK6 | 28 |
| CDK7 | 106 |
| CDK9 | 5.7 |
| CDK13 | 42 |
| CDK17 | 1180 |
| JAK1 | 1190 |
| JAK2 | 957 |
| JAK3 | 451 |
| TYK2 | 111 |
| PDGFRb | 36 |
| Aurora A | 580 |
| FLT3 | 39 |
| MKNK2 | 3430 |
| ROCK2 | 2090 |
Supplementary Table 3. Potencies of KAN04415171C against off-target kinases determined by Flashplate-based radiometric 33P assay (ProQinase, Freiburg, Germany) subsequent to identification of off-target effectors via KINOMEscan™ (DiscoverX, San Diego, CA, USA) profiling.

## Slide 10
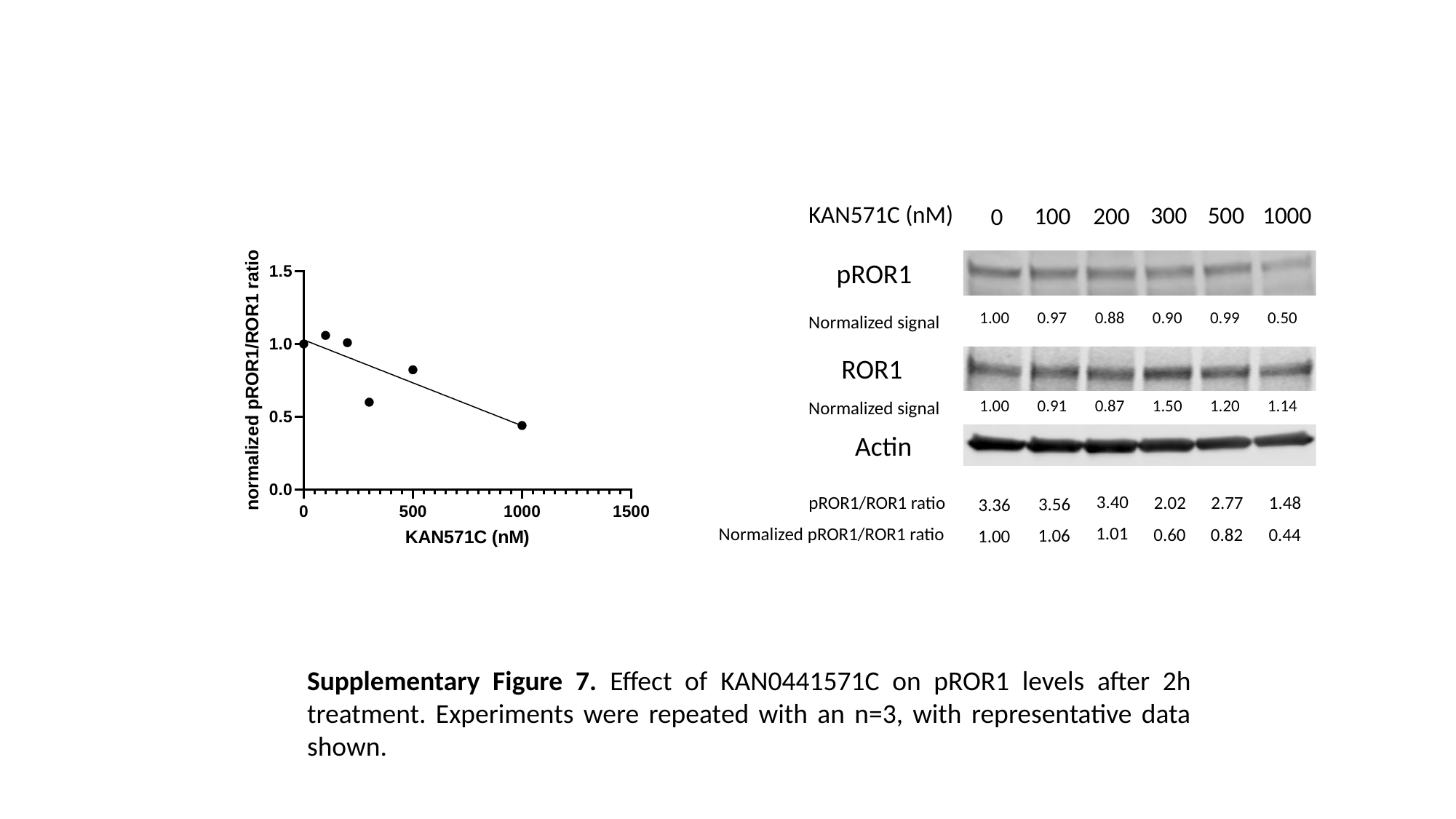

KAN571C (nM)
300
500
1000
100
200
0
pROR1
Normalized signal
| 1.00 | 0.97 | 0.88 | 0.90 | 0.99 | 0.50 |
| --- | --- | --- | --- | --- | --- |
ROR1
Normalized signal
| 1.00 | 0.91 | 0.87 | 1.50 | 1.20 | 1.14 |
| --- | --- | --- | --- | --- | --- |
Actin
3.40
pROR1/ROR1 ratio
2.02
2.77
1.48
3.56
3.36
1.01
Normalized pROR1/ROR1 ratio
0.60
0.82
0.44
1.06
1.00
Supplementary Figure 7. Effect of KAN0441571C on pROR1 levels after 2h treatment. Experiments were repeated with an n=3, with representative data shown.

## Slide 11
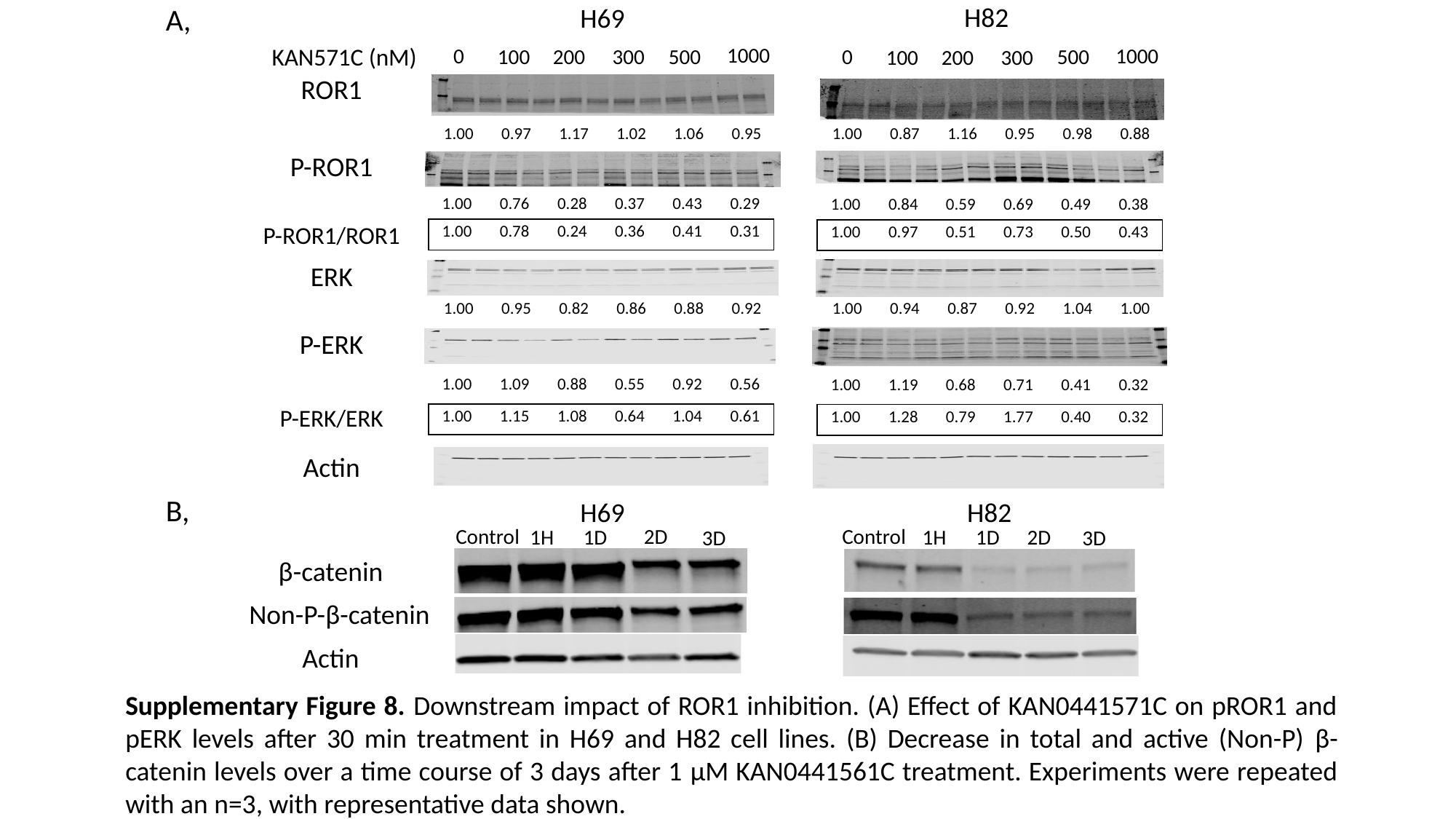

H82
H69
A,
KAN571C (nM)
1000
1000
0
500
0
100
200
300
500
100
200
300
ROR1
| 1.00 | 0.97 | 1.17 | 1.02 | 1.06 | 0.95 |
| --- | --- | --- | --- | --- | --- |
| 1.00 | 0.87 | 1.16 | 0.95 | 0.98 | 0.88 |
| --- | --- | --- | --- | --- | --- |
P-ROR1
| 1.00 | 0.76 | 0.28 | 0.37 | 0.43 | 0.29 |
| --- | --- | --- | --- | --- | --- |
| 1.00 | 0.84 | 0.59 | 0.69 | 0.49 | 0.38 |
| --- | --- | --- | --- | --- | --- |
P-ROR1/ROR1
| 1.00 | 0.78 | 0.24 | 0.36 | 0.41 | 0.31 |
| --- | --- | --- | --- | --- | --- |
| 1.00 | 0.97 | 0.51 | 0.73 | 0.50 | 0.43 |
| --- | --- | --- | --- | --- | --- |
ERK
| 1.00 | 0.95 | 0.82 | 0.86 | 0.88 | 0.92 |
| --- | --- | --- | --- | --- | --- |
| 1.00 | 0.94 | 0.87 | 0.92 | 1.04 | 1.00 |
| --- | --- | --- | --- | --- | --- |
P-ERK
| 1.00 | 1.09 | 0.88 | 0.55 | 0.92 | 0.56 |
| --- | --- | --- | --- | --- | --- |
| 1.00 | 1.19 | 0.68 | 0.71 | 0.41 | 0.32 |
| --- | --- | --- | --- | --- | --- |
P-ERK/ERK
| 1.00 | 1.15 | 1.08 | 0.64 | 1.04 | 0.61 |
| --- | --- | --- | --- | --- | --- |
| 1.00 | 1.28 | 0.79 | 1.77 | 0.40 | 0.32 |
| --- | --- | --- | --- | --- | --- |
Actin
B,
H69
H82
Control
Control
2D
2D
1H
1H
1D
1D
3D
3D
β-catenin
Non-P-β-catenin
Actin
Supplementary Figure 8. Downstream impact of ROR1 inhibition. (A) Effect of KAN0441571C on pROR1 and pERK levels after 30 min treatment in H69 and H82 cell lines. (B) Decrease in total and active (Non-P) β-catenin levels over a time course of 3 days after 1 μM KAN0441561C treatment. Experiments were repeated with an n=3, with representative data shown.

## Slide 12
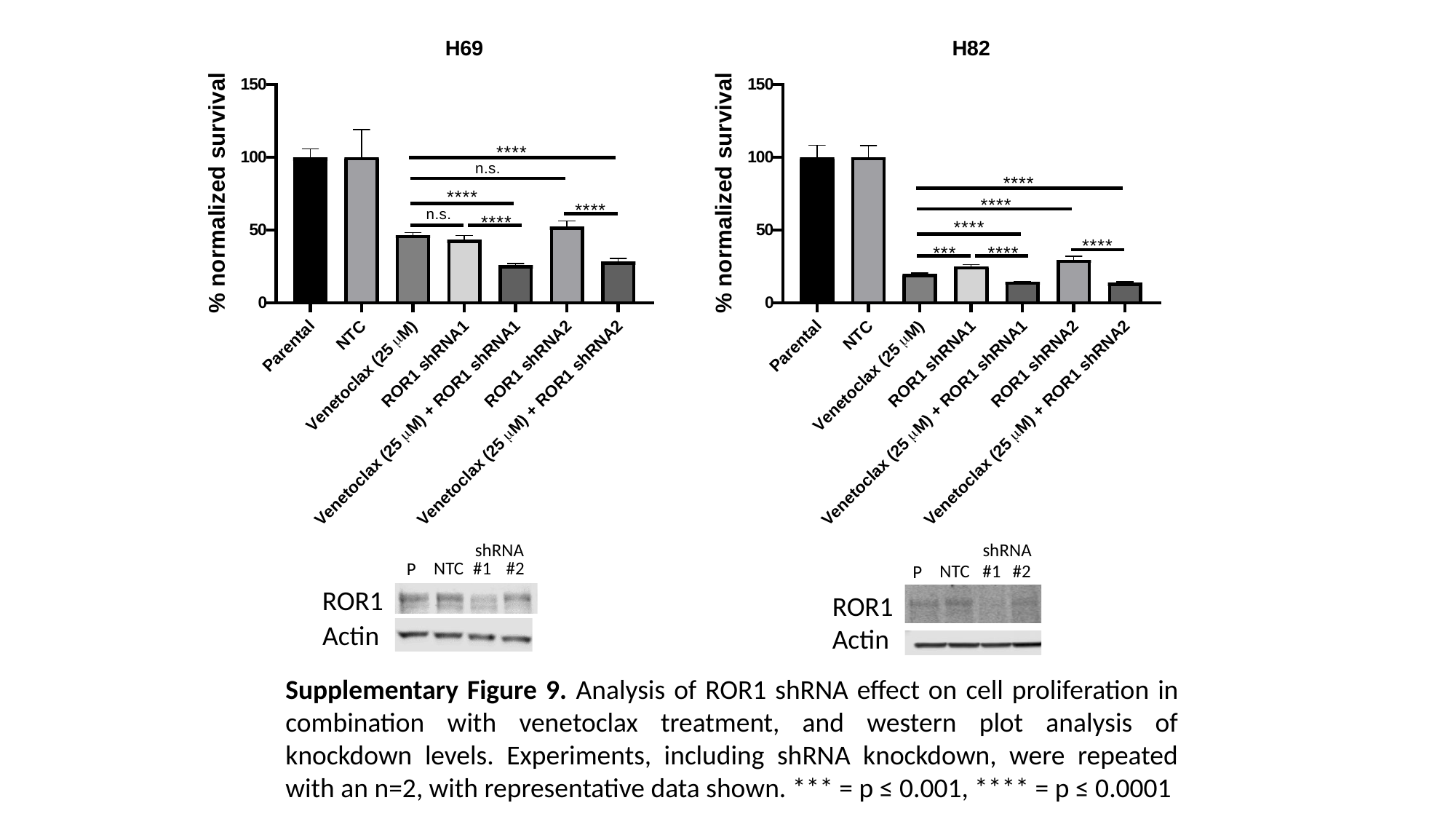

shRNA
NTC
P
ROR1
Actin
#1
#2
shRNA
NTC
P
ROR1
Actin
#1
#2
Supplementary Figure 9. Analysis of ROR1 shRNA effect on cell proliferation in combination with venetoclax treatment, and western plot analysis of knockdown levels. Experiments, including shRNA knockdown, were repeated with an n=2, with representative data shown. *** = p ≤ 0.001, **** = p ≤ 0.0001

## Slide 13
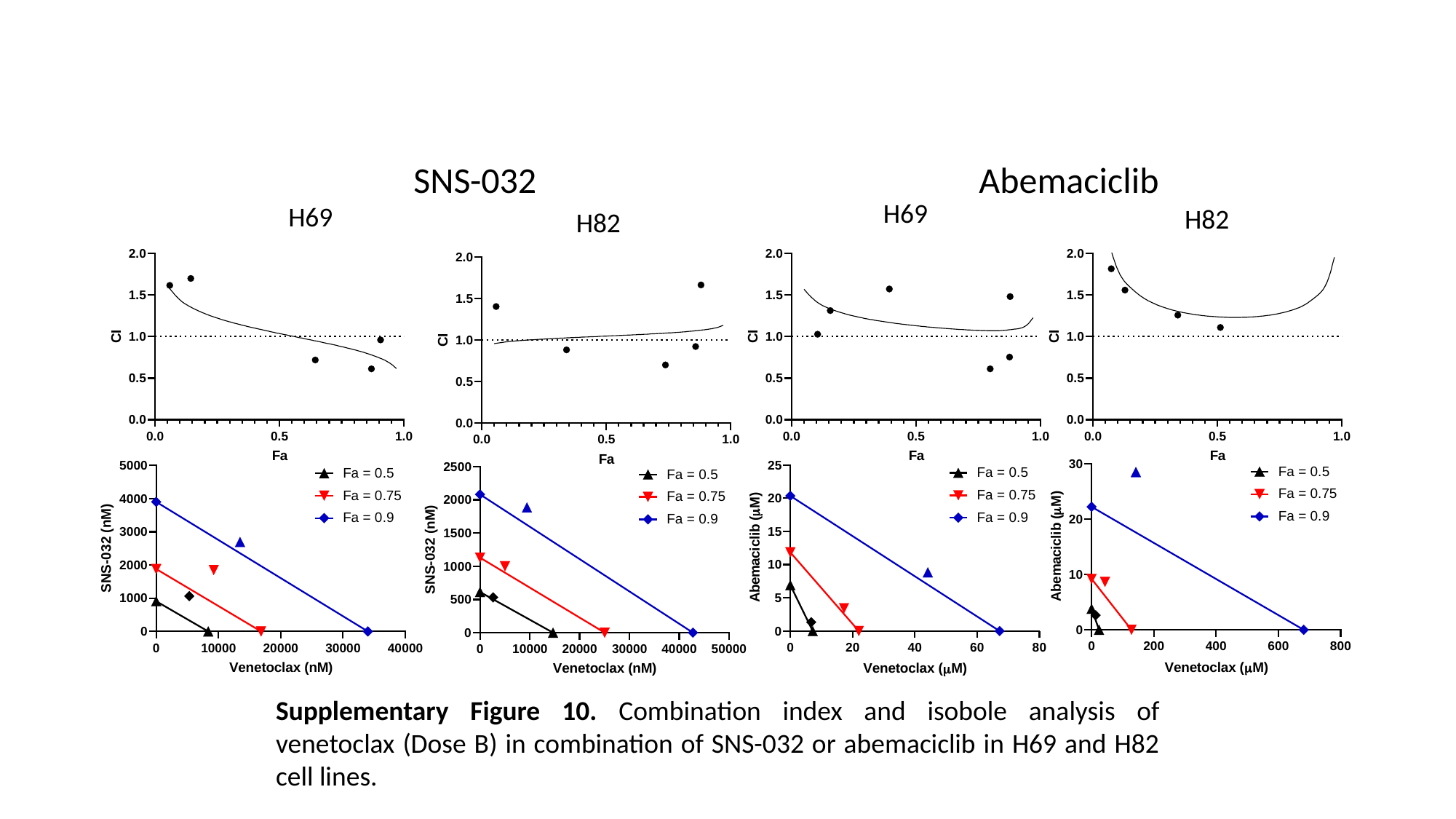

SNS-032
Abemaciclib
H69
H69
H82
H82
Supplementary Figure 10. Combination index and isobole analysis of venetoclax (Dose B) in combination of SNS-032 or abemaciclib in H69 and H82 cell lines.
